# Prenatal Vitamin B12 and Children’s Brain Development and Cognitive, Language and Motor Outcomes: A Scoping Review

**DOI:** 10.3390/children11050558

**Published:** 2024-05-07

**Authors:** Fasika Jembere, Deborah Dewey

**Affiliations:** 1 Undergraduate Medical Education, Cumming School of Medicine, University of Calgary, Calgary, AB T2N 4N1, Canada; fasika.jembere1@ucalgary.ca; 2 Department of Pediatrics, Cumming School of Medicine, University of Calgary, Calgary, AB T2N 1N4, Canada; 3 Owerko Centre, Alberta Children’s Hospital Research Institute, University of Calgary, Calgary, AB T2N 1N4, Canada; 4 Department of Community Health Sciences, Cumming School of Medicine, University of Calgary, Calgary, AB T2N 1N4, Canada; 5 Hotchkiss Brain Institute and Mathison Centre for Mental Health and Research, University of Calgary, Calgary, AB T2N 4N1, Canada

**Keywords:** vitamin B12, prenatal supplementation, maternal, brain development, cognitive outcomes

## Abstract

Adequate maternal nutrient intake of vitamin B12 is critical to fetal brain development and subsequent neurodevelopmental outcomes. We conducted a scoping review to map the current state of knowledge from human epidemiological studies on the associations between maternal vitamin B12 during pregnancy and children’s brain, cognitive, language, and motor development to identify gaps in the literature and suggest directions for future research. PubMed and OVID MEDLINE were searched. Search terms were vitamin B12, prenatal or maternal, neurodevelopment or cognitive development or brain. Animal studies were excluded. In total, 148 publications were identified, of which 19 met our inclusion criteria: (1) maternal vitamin B12 assessed via a measure of status, dietary intake, supplementation, or deficiency; and (2) an outcome related to brain development or cognitive, language, or motor development in children less than 18 years of age was assessed. This scoping review suggests that evidence supporting a relationship between maternal vitamin B12 during pregnancy and children’s neurodevelopmental outcomes is inconclusive. Further longitudinal research is needed to clarify the effects of maternal vitamin B12 supplementation, status, and intake on children’s brain development and neurodevelopmental outcomes.

## 1. Introduction

Pregnancy is a time of rapid physiological and metabolic changes. Nutritional requirements increase during pregnancy and adequate maternal nutrition plays a key role in maintaining a healthy pregnancy. In utero, the developing brain is sensitive to concentrations of maternal micronutrients [1] and maternal nutrition is critical for fetal brain development and subsequent neurodevelopmental outcomes [2]. Brain development begins about 2 weeks after conception. The process of neurulation is under genetic control but can be influenced by the prenatal maternal environment. For example, toxins (e.g., alcohol) or nutrient deficiencies (e.g., folate) can negatively impact fetal brain development both in the short and long-term [3]. In the early fetal period, brain development is rapid and certain processes such as neural cell proliferation and cell migration occur only during gestation. Other neural changes such as synapse formation and neurogenesis continue into childhood. These neural processes are optimized by adequate maternal nutrient intake during the prenatal period.

During pregnancy, it is recommended that certain macronutrients and micronutrients are consumed in adequate amounts, in other words, not in excess or deficiency, to support optimal fetal development and subsequent neurodevelopment [4]. Health Canada’s *Prenatal Nutrition Guidelines for Health Professionals* provides recommended dietary allowances for select nutrients including vitamin B12, folate, and iron, all of which are essential to fetal development [5,6]. Research has also reported associations between lower maternal nutrient concentrations of vitamin B12, folate, choline, iron, vitamin D, and polyunsaturated fatty acids, and poorer neurodevelopmental outcomes in children [1,7,8].

Vitamin B12 is a critical micronutrient that supports the rapidly changing metabolic demands of pregnancy and is involved in various metabolic pathways. Deficiencies in vitamin B12 during pregnancy have been related to adverse maternal outcomes such as obesity, insulin resistance, hyperglycemia, and dyslipidemia [9,10] and these disorders can have an adverse impact on fetal development. Moreover, vitamin B12 is a methyl-donor nutrient, which is vital to the fetal development process [11]. The development and normal function of the central nervous system is dependent on methylation reactions that involve the donation of methyl groups by S-adenosylmethionine (SAM) to various substrates. The production of SAM requires methionine, which partly depends on the maternal dietary intake of methyl-donor nutrients, which include vitamin B12 and folate [12]. These methyl-donor nutrients support several neural processes including neurogenesis, myelination, and synaptic plasticity [2]. 

Prenatal nutrient deficiencies in vitamin B12 can interfere with early brain development and restrict essential processes such as myelination and synaptic connectivity [13,14]. Myelination is a fundamental physiological process that begins in the second trimester of pregnancy and continues through the second year of life, and into early adolescence and adulthood [15,16]. A case study conducted on a 14 ½-month-old child with anemia associated with insufficient maternal intake of vitamin B12 found on MRI severe brain atrophy and demyelination [17]. Thus, prenatal vitamin B12 deficiencies during the gestation period could have significant consequences on central nervous system development and function [18]. 

Neurodevelopment refers to the brain’s development of neurological pathways that influence intellectual functioning, language, motor skills, vision and hearing, learning, attention, memory, and behavior. Cognition is a more circumscribed term that refers to a complex set of higher-order brain functions including attention, memory, language, perception, and learning [19,20]. Cognitive, language, and motor functions play a vital role in development and are key to an individual’s long-term physical and mental health, academic success, and economic outcomes. 

Neurodevelopment is influenced by many factors and there is increasing evidence in the research literature that supports connections between adequate maternal B12 during pregnancy, optimal brain development in offspring, and better neurodevelopmental outcomes. However, human studies that have investigated associations between maternal B12 status or intake and children’s cognitive, language, and motor outcomes have reported inconsistent findings. Cruz-Rodríguez found that medium levels of maternal vitamin B12 (i.e., 312 to 408 pg/mL) in the first trimester of pregnancy were associated with higher scores on the Bayley-III cognitive, motor, and language domains at 40 days after birth compared to low levels of vitamin B12 [21]. In the Growing Up in Singapore Towards Healthy Outcomes (GUSTO) cohort, Lai et al. reported that the children of mothers with blood concentrations of vitamin B12 that were deficient had lower scores on the cognitive composite of the Bayley Scales of Infant Development—Third Edition (BSID-III) at 24 months of age [22]. No significant associations were found for the BSID-III language and motor domains. In another cohort study, Wu et al. found no significant associations between maternal plasma vitamin B12 concentrations and infant cognitive, language, or motor outcomes on the BSID-III [23]. Randomized controlled trials (RCTs) have also reported inconsistent findings with two studies finding no significant association of maternal vitamin B12 supplementation [24,25], on infants’ cognition, language and motor outcomes measured with BSID-III and one study reporting significant effects for only expressive language [26]. Thus, the evidence to date on maternal vitamin B12 and children’s cognitive, language, and motor outcomes appears to be inconclusive. 

To better understand the state of the epidemiological evidence in human populations on the associations between maternal vitamin B12 concentrations during pregnancy and neurodevelopmental outcomes in children, we undertook a scoping review. The aim of this review was to map the current state of knowledge, identify gaps in the literature, and suggest directions for future research. 

## 2. Methods

**Figure 1:**
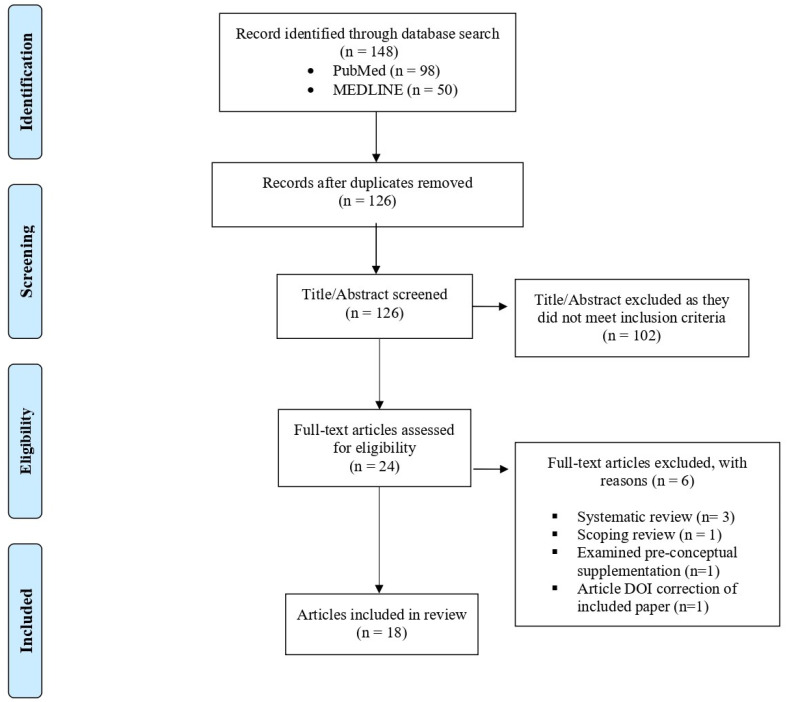
PRISMA flow diagram of inclusion process.

The proposed scoping review was conducted following the JBI methodology for scoping reviews [27]. Prior to conducting the review, a protocol was developed, which identified the review questions, and inclusion criteria (population, exposure of interest, outcomes, type of studies). Our *review questions* were as follows: (1) What is the current state of knowledge on the associations between maternal vitamin B12 concentrations during pregnancy and children’s brain, and cognitive and motor development? (2) Are there gaps in the literature? (3) What future research is needed? The *population of studies* included in the review were all human studies that investigated associations between maternal vitamin B12 status, dietary intake, supplementation, or deficiency, and child brain or cognitive or motor development. Our *exposure of interest* was maternal vitamin B12 status, dietary intake, supplementation, or deficiency. Our *primary outcomes* were child brain development and child cognitive, language, or motor outcomes in individuals less than 18 years of age. Randomized controlled trials, prospective and retrospective cohort studies, case–control studies, and analytic cross-sectional studies were considered in the review. Single-case designs, case studies, qualitative studies, and protocol studies were excluded. We searched PubMed and OVID MEDLINE on the 29 June 2023. The search strategy was developed with assistance from a medical librarian and targeted journal titles and abstracts. There was no restriction on publication date, study location, or language of the publication. Search terms consisted of vitamin B12, prenatal or maternal, neurodevelopment or cognitive development or brain. Animal studies were excluded. Covidence was used to import the references. The initial search produced 148 publications; in total, 22 duplicates were removed. FJ and DD screened the titles and abstracts of 126 articles to ensure that they met our inclusion criteria. Any conflicts regarding inclusion were resolved through discussion. After, initial screening 24 articles were identified for a full text review. Following the full text review, 19 articles were retained for inclusion in the scoping review. Papers were retained if they had maternal vitamin B12 as an exposure of interest, either through measures of status, dietary intake, supplementation, or maternal deficiency. Only studies that reported outcomes of brain development or cognitive or motor development in children less than 18 years of age were retained. Studies that did not measure maternal vitamin B12 prenatally or did not report outcomes related to brain or cognitive or motor development in the offspring were excluded (see Figure 1).

## 3. Results

All studies included in this scoping review examined associations between maternal vitamin B12 intake or supplementation and child neurodevelopmental outcomes including intelligence, language, motor skills, memory, executive function, and social development. One study also investigated associations between vitamin B12 and child brain development [28]. Of the studies that met our inclusion criteria, 3 were randomized controlled trials, 11 were prospective cohort studies, and 2 were retrospective cohort studies. Among the prospective cohort studies, some studies used data from the same cohort: two studies reported on data from the PUNE cohort, and three studies used data from the ALSPAC cohort. The characteristics of the studies are summarized in Table 1.

Although we placed no limitations on the date of publication, all the studies identified were published between 2008 and 2023. The studies were demographically diverse and included publications from Spain, the United States, India, Kenya, Singapore, the United Kingdom, the Netherlands, Nepal, Canada, and Mexico. There were large variations in sample sizes, which ranged from 98 to 6667 mother–child pairs. Some studies identified specific exclusion criteria (i.e., history of diabetes before pregnancy, adverse obstetric history, multiple or abnormal pregnancies, any chronic medical conditions), while other studies focused on specific populations (i.e., mothers with epilepsy, children diagnosed with autism). There was a large variation in the age at which children’s neurodevelopment was assessed ranging from as young as 72 h postpartum to 15 years of age. One study did not indicate the age at which children were assessed [29]. 

**Table 1:** Characteristics of human studies that examined associations between maternal vitamin B12 during pregnancy and children’s neurodevelopment.

	Study Design	Location	Participants	Method Used to Assess Prenatal Vitamin B12 Level/Method of Supplementation	Time Maternal Vitamin B12 Assessed	Vitamin B12 Intake/Vitamin B12 Status/Vitamin B12 Supplement Dose	Child Age at Assessment	Assessment Measures
**Studies with Associations**								
Bhate et al., 2012 [30]	Prospective Cohort Study; (Pune Maternal Nutrition Study; PUNE)	India	123 mother–child pairs	Maternal blood samples; Semi-quantitative food frequencyquestionnaire (FFQ)	Blood sampled at 28 & 34 weeks of gestation	Sufficient vitamin B12 defined as >150 pmol/L	2 years	Developmental Assessment Scale for Indian Infants (DASII); Vineland Social Maturity Scale
Bhate et al., 2008 [31]	Prospective Cohort Study (Pune Maternal Nutrition Study; PUNE)	India	118 mother–child pairs	Maternal blood samples	Blood sampled at 28 weeks of gestation	Low plasma vitamin B12 concentrations defined as <77 pmol/L; High plasma vitamin B12 defined as >224 pmol/L	9 years	Raven’s Colored Progressive Matrices (Raven’s CPM); Visual Recognition; Color Trail Test (CTT); Digit Span Test
Bonilla et al., 2012 [32]	Prospective Cohort Study (Avon Longitudinal Study of Parents and Children ALSPAC)	United Kingdom	4787 mother–child pairs	FFQ	FFQ completed at 32 weeks of gestation	Not defined	8 years	Wechsler Intelligence Scale for Children-3rd Edition (WISC-III)
Cruz-Rodríguez et al., 2023 [21]	Prospective Cohort Study (The ECLIPSES study)	Spain	434 mother–child pairs	Maternal blood samples	Blood sampled at 12 and 36 weeks of gestation	Vitamin B12 levels (pg/mL) divided into tertiles: low defined as (<312 pg/mL;medium defined as 312–408 pg/mL;) high defined as ≥409 pg/mL	40 days postpartum	Bayley Scales of Infant and Toddler Development-3rd Edition (BSID-III)
del Río Garcia et al., 2009 [33]	Prospective Cohort Study	Mexico	253 mother–child pairs	Semi-quantitative FFQ	FFQ completed in the first trimester of pregnancy	Low vitamin B12 intake defined as <2 µg/day	1, 3, 6, & 12 months	Bayley Scales of Infant and Toddler-2nd Edition (BSID-II) Spanish version
Golding et al., 2021 [34]	Prospective Cohort Study (Avon Longitudinal Study of Parents and Children; ALSPAC)	United Kingdom	6667–4778 mother–child pairs; the number of children who completed neurodevelopmental assessments varied by age	FFQ	FFQ completed at 32 weeks of gestation	Mean intake 4.87 µg/day; Median intake 4.27 µg/day; Intake below the 10th percentile of the distribution was classified as low (i.e., <2.264 µg/day)	Between 2 and 15 years	Wechsler Intelligence Scale for Children-3rd Edition (WISC-III); Wechsler Abbreviated Scale of Intelligence (WASI); Speech & Language Measures (vocabulary, word combination, intelligibility); Spelling; Reading (word reading, reading speed); Math (mathematical reasoning tests, mental arithmetic); Science (scientific reasoning)
Lai et al., 2019 [22]	Prospective Cohort Study; (Growing Up in Singapore Towards Healthy Outcomes; GUSTO)	Singapore	443 mother–child pairs	Maternal blood samples	26–28 weeks of gestation	Deficient (<148 pmol/L); Insufficient (148 to 221 pmol/L);Sufficient (≥221 pmol/L)	24 months	Bayley Scales of Infant and Toddler Development-3rd Edition (BSID-III)
Neumann et al., 2013 [35]	Prospective Cohort Study (Kenya Project: Nutrition Collaborative Research Support Program)	Kenya	98 mother–child pairs	Prenatal food intake was measured monthly using a quantitative weighing method (i.e., the food prepared and consumed by the mother was measured by weight and volume) and a dietary recall method; food intake was recorded for 2 consecutive days/month	Women enrolled in the first or second trimester of pregnancy and followed to 6 months postpartum	Low vitamin B12 intake was defined as <500 pg/mL	Within 72 h of birth	Brazelton Neonatal Behavioral Assessment Scale (BNBAS)
Raghavan et al., 2018 [29]	Retrospective Cohort Study (The Boston Birth Cohort)	United States	1257 mother–child pairs	Maternal blood samples	24–72 h post-delivery	Lowest vitamin B12 concentrations (<10 percentile; <247 pmol/L) and highest vitamin B12 concentrations (>90th percentile, ≥536.8 pmol/L > deciles) were compared to concentrations that fell in the range of >247 but <536.8	Not identified	Diagnosis of autism, Asperger syndrome, and/or pervasive developmental obtained from the electronic medical record
Thomaset al., 2019 [26]	Randomized Control Trial (Parent Randomized Controlled Trial)	India	218 mother–child pairs	Maternal daily vitamin B12 supplementation from 14 weeks of gestation to 6 weeks postpartum	--	B12 Supplementation (50 µg/day); Placebo	30 months	Bayley Scales of Infant and Toddler Development-3rd Edition (BSID-III)
Veena et al., 2010 [36]	Prospective Cohort Study (Mysore Parthenon Birth Cohort)	India	536 mother–child pairs	Maternal blood samples	30 ± 2 weeks of gestation	Low vitamin B12 status was defined as concentrations <150 pmol/L	9–10 years	Kaufman Assessment Battery for Children (KABC)
Villamor et al., 2012 [12]	Prospective Cohort Study (Project Viva)	United States	1210 mother–child pairs	Maternal self-report 166-item semi-quantitative food FFQ;slightly modified for use in pregnancy	1st trimester and 2nd\trimester of pregnancy	Average daily intake (µg/day) estimated from the FFQs	3 years	Peabody Pictures Vocabulary Test III (PPVT-III); WideRange Assessment of Visual Motor Abilities(WRAVMA)
**Studies with No Associations**								
Ars et al., 2016 [28]	Prospective Cohort Study	Netherlands	244 mother–child pairs	Maternal blood samples	Early pregnancy (mean gestationalage = 13.5 weeks)	__	6–8 years	Snijders-Oomen Niet-VerbaleIntelligence Test; NEPSY-II-NL; Child Behavior Checklist (CBCL); Brain volume outcomes (i.e., total, cortical, and subcortical grey matter, white matter total volumes of the ventricles, thalamus, caudate, putamen, hippocampus)
Caramaschi et al., 2017 [37]	Prospective Cohort Study (Avon Longitudinal Study of Parents and Children; ALSPAC) -Prospective cohort study (Genetics of Overweight Young Adults, GOYA)	United Kingdom; Denmark	Study subsamples: ALSPAC (ARIES), N = 641; ALSPAC (NON-ARIES) N = 3843; GOYA N = 916	Maternal *FUT2* genotype was used as a proxy for circulating B12 levels in pregnancy; the *FUT2* gene has been associated with serum vitamin B12 levels.	--	--	8 years	Abbreviated version of the Wechsler Intelligence Scale for Children-3rd Edition (WISC-III)
Chandyo et al., 2023 [24]	Randomized Control Trial	Nepal	800 mother–child pairs	Maternal supplementation commenced prior to 15 weeks’ gestation and continued to 6 months postpartum	--	B12 supplementation (50 µg/day); Placebo	6 and 12 months	Bayley Scales of Infant and Toddler Development-3rd Edition (BSID-III)
Sadat-Hossieny et al., 2021 [38]	Retrospective Cohort Study Neurodevelopmental Effects of Antiepileptic Drugs (NEAD) study	United States & United Kingdom	305 mother–child pairs	Block 98 FFQ	Throughout pregnancy (not definedat what stage)	--	6 years	Differential Ability Scales (DAS-II)
Srinivasan et al., 2016 [25]	Randomized Control Trial (Parent Randomized Controlled Trial)	India	178 mother–child pairs	Maternalsupplementation commenced at <14 weeks gestations to 6 weeks postpartum	__	B12 supplementation (50 µg/day); Placebo	9 months	Bayley Scales of Infant and Toddler Development-3rd Edition (BSID-III)
Wu et al., 2012 [23]	Prospective Cohort Study	Canada	154 mother–child pairs	Maternal blood samples;FFQ	16 and 36 weeks of gestation	Low vitamin B12 status defined as <148 pmol/L	18 months	Bayley Scales of Infant and Toddler Development-3rd Edition (BSID-III)

The three randomized controlled trials used the same level of supplementation, 50 µg/day [24,25,26]. Further, all three studies commenced supplementation at <15 gestation. However, the length of supplementation varied with two studies providing supplementation to 6 weeks post-partum [25,26] and one to 6 months post-partum [24]. Cohort studies measured prenatal vitamin B12 status in maternal blood samples and/or intake via participants’ responses on food frequency questionnaires (FFQ) collected at different weeks of gestation. In some studies, maternal vitamin B12 was measured in the first trimester [2,12], while in others, it was measured later in pregnancy [21,22,28,30,31,34]. A retrospective cohort study used maternal blood collected 24–72 h post-delivery as a proxy measure of maternal B12 levels during pregnancy [29] and one study used maternal *FUT2* genotype as a proxy for circulating B12 levels in pregnancy [37]. 

In studies that examined maternal concentrations of vitamin B12 in blood, quantification of low, sufficient, and high B12 status in pregnancy differed. The lowest threshold used to define low vitamin B12 was <77 pmol/L [31]. Those studies that assessed vitamin B12 intake using FFQs typically defined low vitamin B12 as <2.0 µg/day or <2.6 µg/day. Some studies did not provide thresholds for low intake or status but investigated associations using continuous estimates of B12 concentrations [28,32,37,38]. 

Standardized measures were used to assess children’s neurodevelopment outcomes, but the measures used varied based on the ages of the children. The Bayley Scales of Infant and Toddler Development were used in seven studies to assess neurodevelopmental outcomes in children aged 1 to 30 months of age [21,22,23,24,25,26,33]. In older children, neurodevelopment was assessed using various measures including the Wechsler Intelligence Scale for Children—Third Edition (WISC-III) [32,34,37], Differential Abilities Scale—Second Edition (DAS-II) [38], the Kaufman Assessment Battery for Children (KABC) [36], and the Peabody Picture Vocabulary Test—Third Edition (PPVT-III) [12]. 

The findings of the studies included in this review are summarized in Table 2. Of the three randomized controlled trials, only Thomas et al. [26] found that B12 supplementation improved cognitive outcomes. They reported that oral vitamin B12 supplementation resulted in higher expressive language scores on the BSID-III in children at 30 months of age. Vitamin B12 supplementation was not associated with improved BSID-III cognitive or motor outcomes. Thomas et al. [35] noted that the intervention and placebo groups were comparable in terms of demographic and biochemical characteristics, but in the placebo group, there were more women with B12 deficiency (N = 61, 58.6%) compared to the intervention group (N = 44, 38.6%). Chandyo et al. [24] found no effect of maternal B12 supplementation on child cognitive, language, or motor outcomes on the BSID-III at 5 and 12 months of age. They measured baseline maternal B12 status prior to supplementation and found that 569 (71%) of 800 eligible pregnant women in the trial had a low or marginal status (<221 pmol/L) [35]. Srinivasan et al. [25] also reported that supplementation was not associated with child outcomes on the BSID-III at 9 months of age. They measured B12 status at baseline and found that 50–60% of the women had low concentrations of B12 (<150 pmol/L); no differences in baseline B12 status were found between the intervention and placebo group [37].

Eight of the prospective cohort studies reported that low or deficient maternal vitamin B12 status or intake was associated with poorer neurodevelopment outcomes, including lower intelligence, and poorer speech and language development, gross motor skills, and social development [21,22,25,30,31,32,33,34]. Outcomes were examined in children as young as 72 h post-delivery to 15 years of age. Two studies reported that higher maternal vitamin B12 intake [12] or status [12,36] was associated with poorer language outcomes, specifically receptive language and verbal fluency, respectively. Two prospective cohort studies that examined child cognitive outcomes at 18 months or 6 to 8 years reported no associations between maternal vitamin B12 status and child cognitive outcomes [23,28]. Two retrospective cohort studies were identified in this scoping review. One reported that elevated maternal plasma vitamin B12 (>600 pmol/L) in a maternal blood sample acquired 24–72 h post-delivery was associated with an increased risk of autism spectrum disorder (ASD) [29]. The second retrospective cohort study examined whether vitamin B12 intake in mothers with epilepsy was associated with their children’s neurodevelopment; no associations were found [38]. 

Two studies used data from the Avon Longitudinal Study of Parents and Children (ALSPAC) and other longitudinal cohort studies to investigate potential genetic or epigenetic factors that could influence maternal B12 concentrations and child cognitive outcomes. Bonilla et al. [32] reported a positive association between maternal B12 intake and child intelligence. They also examined the association between genes that are involved in the transport of vitamin B12, *FUT2* and *TCNS*, and child intelligence [32]. Results revealed that an increasing number of *FUT2* alleles in the mothers was weakly associated with child intelligence. Caramaschi et al. [37] examined the potential mediating role of DNA methylation in the relationship between maternal B12 and child intelligence, using maternal *FUT2* genotype as a proxy for circulating B12 levels. They found little evidence to support a causal effect of increasing maternal vitamin B12 on child intelligence. 

Support for the potential influence of trimester-specific measures of B12 status or maternal vitamin B12 intake during a particular trimester in pregnancy on child outcomes was mixed. For example, two prospective cohort studies that assessed maternal B12 status in the first trimester of pregnancy reported positive associations with children’s cognitive, language, motor, and social development [21,33], whereas one reported no association [28]. Studies that examined the influence of higher maternal B12 status during the second trimester did not find any associations with child neurodevelopment [23] or negative associations [12]. Four of the studies that assessed maternal B12 status or intake in the third trimester reported positive associations with child neurodevelopment outcomes [22,30,31,32,34], one reported a negative association [36], and two reported no associations [21,23]. It is notable that Bhate et al. [30] measured vitamin B12 at 24 and 34 weeks of gestation and found a positive association between sufficient maternal B12 levels (>150 pmol) and cognitive and social development at 24 weeks of gestation, but no associations at 34 weeks. Cruz-Rodríguez et al. [21] measured vitamin B12 status at 12 and 36 weeks of gestation, and found that medium maternal vitamin B12 levels (312 to 408 pg/mL and ≥409 pg/mL, respectively) in the first trimester (12 weeks of gestation) were positively associated with higher BSID-III scores on measures of cognition, language, and motor abilities compared to low maternal vitamin B12 (<312 pg/mL). During the third trimester (36 weeks of gestation), no significant associations were observed.

## 4. Discussion

The current evidence on the relationship between prenatal vitamin B12 and children’s neurodevelopmental outcomes is limited. This scoping review identified 18 studies, including 3 randomized controlled trials, 13 prospective cohort studies and 2 retrospective cohort studies, that investigated the influence of maternal vitamin B12 on children’s neurodevelopmental outcomes, and only 1 of these studies investigated associations between maternal vitamin B12 and brain volumes [28]. Overall, the findings are mixed and further research is needed to clarify the effects of maternal vitamin B12 supplementation, status, and intake on children’s brain development and neurodevelopmental outcomes. 

The three RCTs conducted in India or Nepal reported few effects of maternal B12 supplementation during pregnancy on children’s neurodevelopment. The only positive association was for expressive language [26]. All three studies measured maternal vitamin B12 status at baseline prior to the intervention and found that most of the women had B12 deficiency [32,35,37]. Thomas et al. [26] adjusted for maternal baseline B12 status in their statistical analyses; however, Chandyo et al. [24] and Srinivasan et al. [25] did not. Chandyo et al. [24] noted that after randomization there were similar percentages of women who had low or marginal B12 in the supplementation group (70%) and the placebo group (73%). It is noteable that these three studies reported that supplementation improved maternal B12 status. Therefore, the lack of significant findings of the RCTs cannot be attributed to no change in maternal vitamin B12 status or maternal B12 being in the ‘normal’ range, prior to the intervention. 

The child neurodevelopmental assessments conducted by Chandyo et al. [24] and Srinivasan et al. [25] were completed at six and 12 months of age or nine months of age, respectively, while Thomas et al. [26] assessed the children at 30 months of age. It is possible that the influence of maternal vitamin B12 supplementation on children’s neurodevelopment is not evident until later in childhood. Alternatively, the BSID-III, which was used to assess neurodevelopmental outcomes in all three RCTs, may not be sensitive enough in the first years of life to identify potentially subtle effects of vitamin B12 supplementation on children’s neurodevelopment. Further, there could be unmeasured confounders, such as socioeconomic factors, that were not controlled for that may have influenced the relationship between maternal vitamin B12 status and child neurodevelopment. The mixed findings of these RCTs suggest that future research with longer periods of child follow-up is needed to determine if vitamin B12 supplementation during pregnancy improves child neurodevelopment. 

Most of the cohort studies included in this scoping review examined the association between vitamin B12 deficiency (i.e., low) versus sufficiency and neurodevelopmental outcomes. The results of most of these studies suggested that deficient maternal vitamin B12 during pregnancy was related to poorer neurodevelopmental outcomes in children across various domains of function including cognitive, language, and motor development. These associations were found at various ages and in participants from a variety of countries, including low-, middle-, and high-income countries. The relative consistency of these findings across child age and geographic location provides support for the contention that ‘sufficient’ maternal vitamin B12 during pregnancy plays a role in children’s neurodevelopment. It is notable that Raghavan et al. [29] reported that elevated levels of plasma vitamin B12 (>600 pmol/L) compared to non-elevated levels (i.e., ≥200 to <600, sufficient) were associated with increased risk of ASD in children and there was no association between low levels of B12 and ASD risk. Few studies have examined the influence of elevated maternal B12 on child neurodevelopment; however, future research should examine not only the effect of maternal vitamin B12 deficiency on children’s neurodevelopmental outcomes but also elevated levels. 

Various standardized cognitive assessment tools were used to assess children’s neurodevelopment including the BSID-III, WISC-III, KABC, DAS-II, and the NEPSY-II-NL. These measures examine children’s performance across several neurodevelopmental domains at various ages. The findings of some of the studies included in this review suggest that prenatal vitamin B12 status and intake may be associated with overall cognitive functioning, and with very specific domains of function such as working memory (i.e., Digit Span Backward). Future research may want to investigate the specific domains of function associated with deficient maternal vitamin B12 and if the domains affected differ with the age of the child. 

Overall, research examining the relationship between maternal vitamin B12 and child neurodevelopment is scarce and significant gaps exist. Randomized controlled trials that follow children from birth to beyond 30 months of age are needed to determine if maternal B12 supplementation during pregnancy has long-term effects on child outcomes. Further, prospective cohort studies investigating the associations between maternal vitamin B12 status and intake at different stages of pregnancy and children’s neurodevelopmental outcomes are needed. Cruz-Rodríguez et al. [21] and Bhate et al. [30] measured vitamin B12 concentrations at two different points in pregnancy and found positive associations between sufficient maternal vitamin B12 levels and neurodevelopmental outcomes earlier in pregnancy (12 and 28 weeks of gestation, respectively) but not later in gestation (34 and 36 weeks of gestation, respectively). It is possible that sufficient maternal vitamin B12 early in pregnancy may have a greater impact on children’s neurodevelopment outcomes.These findings support the conduct of research that specifically investigates the influence of pre-conceptual supplementation and supplementation during the first trimester on children’s neurodevelopment is needed. More research that examines vitamin B12 levels across multiple time points in pregnancy is also needed to determine if there are critical times during gestation when adequate vitamin B12 is essential to children’s neurodevelopment. 

Most of the studies identified assessed children’s neurodevelopment at a single point in time. Further, in many of the studies, the children were quite young at the time of assessment, infants, or preschoolers. To better quantify the long-term impact of maternal vitamin B12 on children’s neurodevelopment, prospective longitudinal studies need to be conducted that follow children from birth to adulthood, mapping their neurodevelopment across various domains of function over time. This will allow us to determine if maternal vitamin B12 impacts neurodevelopmental domains of function differently as children mature.

Our scoping review had several strengths including no restrictions on publication data, language, and location. This allowed us to examine the association between maternal vitamin B12 and neurodevelopmental outcomes from a global perspective and to include data from low-, middle-, and high-income countries. Additionally, many of the studies included in the review were conducted in the past 10 years demonstrating the evolving interest in the impact of vitamin B12 on children’s neurodevelopmental outcomes. This scoping review provides an overview of the burgeoning data and suggests necessary next steps needed to strengthen our understanding of the influence of maternal vitamin B12 on children’s neurodevelopment. One limitation of the studies published to date is the different operational definitions used to classify maternal status as deficient, sufficient and high. As a result, it is difficult to directly compare the findings of the various studies. Furthermore, much of the research that has examined the neurodevelopmental effects of vitamin D supplementation or deficiency has followed children in only infancy or early childhood. Longer follow-up studies are needed to determine if supplementation or deficiencies are associated with other cognitive processes such as attention, memory or executive function. Another limitation of research to date is the lack of studies investigating associations between maternal B12 status and levels and brain structure and function. We identified only one study that investigated the association between maternal vitamin B12 and brain volumes using brain imaging technologies [28]. Future research using brain imaging modalities is important to elucidate the role of vitamin B12 in brain development. A primary limitation of the study was that we searched two databases (PubMed, OVID Medline) only. A more extensive search of the published and grey literature could have revealed additional studies for inclusion in this review.

## 5. Conclusions

Vitamin B12 is a methyl-donor nutrient that is vital to the fetal brain development [39]. It is required to produce SAM, which needs methionine, an enzyme that is dependent on the maternal dietary intake of methyl-donor nutrients such as B12 [12]. Adequate amounts of vitamin B12 are essential to neurological processes including myelination and synaptic connectivity [13,14]. Therefore, nutritional deficiency in vitamin B12 could adversely affect fetal brain development [40] and in turn the development of cognitive, language, and motor processes. However, the evidence supporting a relationship between maternal vitamin B12 status or intake during pregnancy and children’s neurodevelopmental outcomes is inconclusive. Longitudinal epidemiological studies and RCTs are needed to clarify the effects of maternal vitamin B12 supplementation, status, and intake on children’s brain development and neurodevelopmental outcomes. 

## Figures and Tables

**Table 2:** Results of human studies that examined associations between maternal vitamin B12 during pregnancy and children’s neurodevelopment.

	Results
**Studies with Associations**	
Bhate et al., 2012 [30]	- Mental development at 2 years on the DASII was positively associated with maternal B12 at 28 weeks’ gestation (*r* = 0.19, *p* = 0.041). - Mean standard scores on Mental development in children of mothers with sufficient B12 (>150 pmol/L) at 28 weeks’ gestation were higher than those of children of B12 deficient mothers (101 vs. 98, *p* = 0.035). - Social development at 2 years on the Vineland Social Maturity Scale was positively associated with sufficient maternal B12 levels (>150 pmol/L) at 28 weeks of gestation. - Mean standard scores on Social development in children of B12 sufficient mothers (>150 pmol/L) were higher than those of children of B12-deficient mothers (93 vs. 91, *p* = 0.029).
Bhate, 2008 [31]	- Children with low vitamin B12 (i.e., <77 pM) performed slower on the Color Trail A Test (182 vs. 159 s; *p* = 0.02) and worse on the Digit Span Backward test (number of digits, 2.6 vs. 2.9; *p* = 0.02). - No group differences were found on other tests of cognitive function: Raven’s Color Progressive Matrices, Visual Recognition Color Trail B or Digit Span Backward.
Bonilla et al., 2012 [32]	- Positive association found between maternal vitamin B12 intake and child IQ, 2.0 (95% CI = 1.3, 2.8); this association was markedly attenuated after adjustment for confounders 0.7 (95% CI = −0.04, 1.4). - Maternal B12 elevating allele *FUT2* was weakly associated with children’s IQ 0.9 (95% CI = 0.1, 1.6).
Cruz-Rodríguez et al., 2023 [21]	- Compared to low maternal vitamin B12 (<312 pg/mL), medium concentrations (312 to 408 pg/mL) in the first trimester of pregnancy were associated with higher BSID-III motor (β = 2.766, 95% CI = 0.029, 5.504), gross motor (β = 0.706, 95% CI = 0.153, 1.260), language (β = 2.199, 95% CI = 0.191, 4.207), and cognitive (β = 0.267, 95% CI = 0.005, 0.048) scores at 40 days postpartum. - The probability of obtaining a neonatal motor (OR = 2.43, 95% CI = 1.38, 4.27), gross motor (OR = 1.98, 95% CI = 1.104, 3.56), and receptive language score (OR = 1.79, 95% CI = 1.07, 3.00) > 75th percentile was significantly higher in children of pregnant women who had medium vitamin B12 levels in the first trimester of pregnancy.
del Río Garcia et al., 2009 [33]	- Deficient dietary intake of vitamin B12 in the first trimester of pregnancy was negatively associated with a mental development throughout the first year of life as measured by the BSID-II (β = −1.6; 95% CI = −2.8, −0.3) but not psychomotor development (β = −1.3; 95% CI = −3.8, 0.3).
Golding et al., 2021 [34]	- Before adjustment for confounders significant associations were found between low maternal B12 intake and full-scale IQ at 8 years (β = −3.34; 95% CI = −4.76, −1.92) and 15 years (β = −7.07; 95% CI = −10.01, −4.12); after adjustment for confounders a trend association remained for Performance IQ at 8 years (β = −1.29; 95% CI = −2.77, 0.20). - In adjusted models, children born to mothers who had a low intake of B12 had lower vocabulary scores at 24 months of age, (β = −3.98 95% CI = −7.93, −0.04), reduced ability in combining words at 38 months of age (β = −0.34 95% CI = −0.70, 0.01), and poorer speech intelligibility at 6 years of age (β = −0.28 95% CI = −0.47, −0.09). - In adjusted models, children born to mothers who had a low intake of B12 had poorer math comprehension skills at 8–9 years (β = −0.27 95% CI = −0.60, 0.05), and 10–11 years (β = −0.77 95% CI = −1.33, −0.20), and overall math skills at 13–14 years (β = −2.28, 95% CI = −3.81, −0.75). - In adjusted models, maternal vitamin B12 intake was not association with Reading or Spelling abilities.
Lai et al., 2019 [22]	- Compared with infants of mothers with sufficient vitamin B12, infants of mothers with deficiency had lower cognitive scores on the BSID-III (β = 0.42, 95% CI = −0.70, −0.14). - No significant associations were observed for the BSID-III language or motor scales.
Neuman et al. 2013 [35]	- Vitamin B12 intake during pregnancy was correlated with higher scores on BNBAS reflex subscale, which was measured within the first 72 h after birth (r = −0.19, *p* = 0.05).
Raghavan et al., 2018 [29]	- Elevated levels of maternal plasma vitamin B12 (> 600 pmol/L) compared to normal levels (>200–<600 pmol/L) were associated with increased risk of ASD in offspring (RR = 3.0, 95% CI = 1.6, 5.7). - Deficient vitamin B12 levels (<200 pmol/L) compared to normal levels (>200–<600 pmol/L) were not associated with ASD risk (HR = 1.9, 95% CI = 0.7, 5.3). - Maternal plasma B_12_ levels in the top 10%, when compared to the middle 80%, were associated with an approximately two and half times increased risk of ASD (RR = 2.5, 95% CI = 1.4, 4.5).
Thomas et al., 2019 [26]	- Expressive language sub-domain scores on the BSID-II were significantly higher in children born to women in the vitamin B12 supplementation group compared to the placebo group (β = 0.14, 95% CI = 0.10, 2.36). - Scores on the BSID-III sub-domains of cognition, receptive language, fine motor, and gross motor were comparable between the children of women in the supplementation and placebo groups.
Veena et al., 2010 [36]	- In adjusted models, low maternal vitamin B12 status (<150 pmol/L) was associated with higher verbal fluency (animals) scores (β = 0.19, 95% CI = 0.01, 0.37). - No associations were found between maternal vitamin B12 and cognitive measures that assessed learning, memory reasoning, visuomotor ability, visuomotor processing speed, attention and concentration.
Villamor et al., 2012 [12]	- A weak, inverse association was found between vitamin B12 intake during the second trimester and PPVT-III, scores; every 2.6 ug/d increment in B12 was associated with a 0.4 point decrease in PPVT-III scores at 3 years of age (β = −0.04, CI 95% = −0.8, −0.1).
**Studies with No Associations**	
Ars et al., 2016 [28]	- No significant associations were found between maternal vitamin B12 and child intelligence, NEPSY-II -NL scores (i.e., Total, Language, Attention, Memory/Learning, Sensory motor, Visuospatial) or CBCL emotional and behavioral problems scores. - No associations were found between vitamin B12 and child brain volume outcomes.
Caramaschi et al., 2017 [37]	- Two-step epigenetic Mendelian randomization was used to investigate the potential mediating role of DNA methylation in the relationship between maternal prenatal vitamin B12 concentrations and child IQ. - The first step suggested that maternal vitamin B12 was casually associated with small differences in DNA methylation in the cord blood of offspring. - The second step found little evidence for a potential causal effect of increasing vitamin B12 concentrations prenatally on child IQ.
Chandyo et al., 2023 [24]	- No significant differences were found between the vitamin B12 supplementation group and the placebo group on the BSID-III cognitive, language, and motor domains at 6 or 12 months of age.
Sadat-Hossieny et al., 2021 [38]	- Maternal vitamin B12 estimated from food intake in women with epilepsy was not associated with children’s IQ scores on the DAS-II at 6 years of age.
Srinivasan et al., 2016 [25]	- No significant differences were found between the maternal vitamin B12 supplementation group and the placebo group on the cognitive, language, and motor domain of the BSID-III in infants at 9 months of age.
Wu et al., 2012 [23]	- No significant correlations were found between maternal plasma vitamin B12 concentrations and infant cognitive, language, and motor outcomes on the BSID-III at 18 months of age.

## Data Availability

Data will be made available upon request. The data are not publicly available due to privacy.
